# Chicago Medical Society

**Published:** 1882-04

**Authors:** 


### Society Reports.

### ARTICLE IX.

CHICAGO MEDICAL SOCIETY.

Stated meeting, March 20, 1882, President E. Ingals in the Chair.

Dr. James G. Kiernan read a paper on

### SIMULATION OF INSANITY BY THE INSANE,

And reported many cases, some of which had acquired some celbrity, and clearly proved that such simulation did not necessarily imply a sound mind, even though the insane feigned for some rational motive.

### THE CASE OF GUITEAU.

As the Doctor had been employed as an expert by the defense in the Guiteau trial, he was asked if there had been any simulation of insanity in that case. He said there was not. Guiteau had based his plea on *his inspiration* and made a rational defense, so that in a strict sense he was not feigning insanity. However, as to the soundness of mind of the prisoner, Dr. Kiernan said that he thought his was a very common case of a form of insanity, monomania, which in his case depended on a congenital malformation of the brain. In a strict sense he had no strongly-marked delusion, but that was a matter of slow growth in such cases. However, the Doctor had not the least doubt that Guiteau shot the President while laboring under a delusion.

While in the Oneida Community the prisoner had claimed many inspirations. Here, in Chicago, he had once made such a claim when defending a client guilty of larceny, addressing the court on the second coming of Christ, and his inspiration; and had to be called to order for contempt of the court.

Many physical peculiarities characterized Guiteau's insanity. His whole skull and face were very asymmetrical. Although a perfect symmetry was rare, ordinary departures were very slight. In Guiteau there was a marked deficiency in the posterior region; a marked difference between the two sides of the face, extending to the pupil; and if he had been brought before physicians at an asylum two years previous he had surely been committed.

There came the question whether in the family itself there had been insanity. One uncle died of insanity, another of softening of the brain. His father was pronounced insane by two physicians. A first cousin was insane. One brother and one sister were epileptics. Another relation suffered from exophthalmic goitre. A pamphlet published by the assassin in 1866 resembled very much the ordinary writing of the insane.

Dr. Kiernan subsequently discussed the manner in which experts are called by the defense as well as by the prosecuting party, and thought that the present mode in this country was as good as any other, though susceptible of a good deal of improvement.

Dr. E. Ingals had often advised attorneys not to rely too much on the testimony of experts, and he asked the lecturer whether insanity was a disgrace to the race? Dr. Kiernan did not undertake to answer the question. H. D. V.

